# Motivational Strategies to Prevent Frailty in Older Adults with Diabetes: A Focused Review

**DOI:** 10.1155/2019/3582679

**Published:** 2019-11-22

**Authors:** J. A. Vaccaro, T. Gaillard, F. G. Huffman, E. R. Vieira

**Affiliations:** ^1^Department of Dietetics and Nutrition, Robert Stempel College of Public Health and Social Work, Florida International University, 11200 SW 8th Street, MMC AHC5 324, Miami, FL 33199, USA; ^2^Nicole Wertheim College of Nursing and Health Sciences, Florida International University, 11200 SW 8th St., MMC AHC3 240, Miami, FL 33199, USA; ^3^Department of Dietetics and Nutrition, Robert Stempel College of Public Health and Social Work, Florida International University, 11200 SW 8th Street, MMC AHC5 326, Miami, FL 33199, USA; ^4^Department of Physical Therapy, Nicole Wertheim College of Nursing & Health Sciences, Florida International University, 11200 SW 8th St., MMC AHC3-430, Miami, FL 33199, USA

## Abstract

The prevalence of diabetes among Americans aged 65 years and older is greater than 25%. Medical expenditures for persons with diabetes are more than twice as high as those for patients without diabetes. Diabetes in older adults often times coexists with frailty, resulting in reduced quality of life and increased health-care use. Many older adults with type 2 diabetes have mobility impairments and experience falls, which contributes to increased frailty. Exercise has a protective effect for frailty and falls, yet less than half of persons with diabetes exercise and approximately one-quarter meet exercise recommendations. In addition to exercise, nutrition may help reduce the risk for falls; however, nutritional interventions have not been tested as a fall-prevention intervention. According to a review, there is insufficient evidence to create nutritional guidelines specific for frail older adults with type 2 diabetes. There is a need to motivate and empower older adults with type 2 diabetes to make lifestyle changes to prevent frailty. The purpose of this review was to identify and integrate what is known and what still needs to be done for this population to be successful in making health behavior changes to reduce frailty. There is some evidence that motivational approaches have worked for older adults with various chronic disease conditions. However, studies applying motivational strategies are lacking for frail older adults with type 2 diabetes. A novel motivational approach was described; it combines aspects of the Health Belief Model and Motivational Interviewing. Intervention studies incorporating this model are needed to determine whether this client-driven strategy can help various racial/ethnic populations make the sustainable health behavior changes of increasing exercise and healthy eating while taking into consideration physiological, psychological, and economic barriers.

## 1. Introduction

Diabetes is a complex, chronic disease that requires medical management with a focus on lifestyle changes to prevent complications [1]. Type 2 diabetes is the most common form of diabetes and accounts for 90–95% of cases. For persons with type 2 diabetes, their insulin is produced but is relatively insufficient and leads to hyperglycemia. Chronic hyperglycemia in uncontrolled diabetes can cause long-term damage and dysfunction, particularly of the peripheral nervous system, eyes, kidneys, heart, and blood vessels [1]. According to the American Diabetes Association (ADA), the prevalence of diabetes among Americans aged 65 years and older was 25.2% in 2018 [2]. Medical expenditures for persons with diabetes are more than twice as high as those for patients without diabetes [3]. The estimated cost of diabetes in the U.S. was $237 billion in 2017 [3]. Diabetes in older adults often times coexists with frailty, resulting in reduced quality of life and increased health-care use.

Frailty affects 32 to 48% of older adults with diabetes, compared with only 5 to 10% of those without diabetes [4]. Frailty is a physiological decline that compromises the ability to deal with life's stressors, making people vulnerable to adverse health outcomes. People presenting three out of the following five phenotypic criteria are classified as frail: low grip strength, low energy, slow walking, low physical activity, and unintentional weight loss [5]. Despite Fried and colleague's frailty phenotype classification, there is considerable disagreement as to the operational definition of frailty, its role in the physical and cognitive domains, and its relationship with aging, disability, and chronic diseases [6].

There is a need to motivate and empower older adults with type 2 diabetes to make lifestyle changes to prevent frailty. The purpose of this review was to identify and integrate what is known and what still needs to be done for this population to be successful in making health behavior changes to reduce frailty. Specifically, the aims were to investigate and describe (1) the mechanisms why persons with type 2 diabetes are susceptible to frailty and falls, (2) the relationship between glycemic control and frailty, (3) exercise and frailty, (4) nutrition and frailty, (5) multimodal interventions for frailty in persons with diabetes, and (6) a novel motivational strategy to prevent frailty in older adults with type 2 diabetes.

## 2. Mechanisms for Frailty and Falls in Persons with Type 2 Diabetes

**Figure 1:**
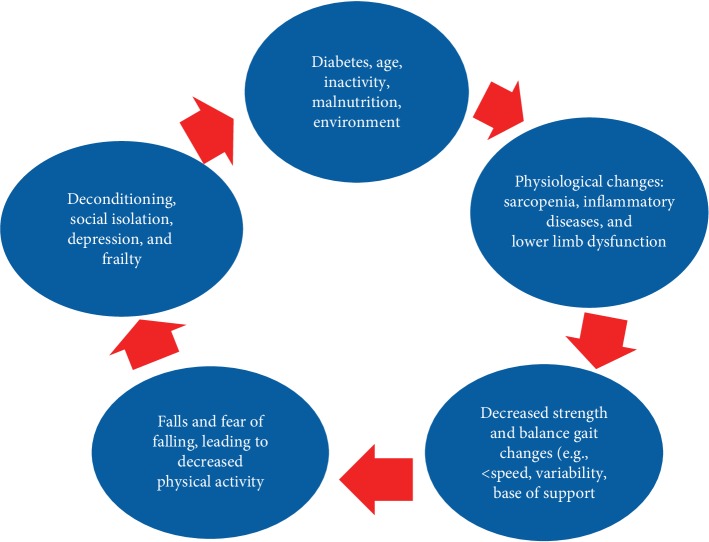
Cycle of changes and factors associated with frailty and falls among older adults with diabetes. Adapted from Vieira et al. [10].

Many older adults with type 2 diabetes have mobility impairments and experience falls [7, 8], which contributes to increased frailty because it creates a vicious cycle of accelerated functional decline and deconditioning (Figure 1). Preventing and reducing diabetes' complications such as peripheral neuropathy, reduced vision, and renal function may help reduce falls [9].

The medical cost for fatal and nonfatal falls for adults aged 65 years and older in the U.S. was $50 billion in 2015, an increase from $38 billion in 2013 [11]. The Center for Disease Control and Prevention (CDC) estimated that fall-related medical costs in 2020 will be $67.7 billion [12, 13] and that a 20% reduction would represent $13.5 billion in savings. The mechanisms predisposing persons with type 2 diabetes and falls are multifacited and have not been clearly determined. Several factors contributed to increased fall risk in a longitudinal study older adults with type 2 diabetes, including reduced renal function, peripheral nerve function, and vision [9]. Stringent glycemic control (A1C <6.0%) leads to hypoglycemial and increased falls [9].

Diabetes was reported as an independent risk factor for falling even after controlling for balance in a prospective study of older European adults [14]. A meta-analysis of community-dwelling older adults aged ≥65 years showed that those who were frail or prefrail had higher rates of falls than those who were robust, and those who were frail were likely to have recurrent falls [15]. Fallers have a 66% chance of suffering subsequent falls within a year [16, 17]. Close to 95% of hip fractures are falls related; 95% of the hip fracture patients are discharged to nursing homes; and 20% die within a year [16, 18]. Falls for persons with diabetes can result in more serious injuries and a longer recovery process as compared with older adults without diabetes [19].

## 3. Glycemic Control and Frailty

Poor diabetes self-management increases functional impairments and diabetes' complications, leading to sarcopenia and frailty [20]. Lack of glycemic control and increase in insulin resistance and/or depletion are associated with loss of muscle mass and strength because insulin is an anabolic hormone [20]. Diabetes causes debilitation across muscle, nerve, and cardiopulmonary and executive reserve systems, leading to frailty and making it increasingly more difficult to maintain glycemic control [20]. Fewest complications were found at A1C levels between 7 and 8 percent [20]. High A1C was associated with heart disease, whereas oral hypoglycemic agents together with malnutrition result in lower than normal A1C levels producing hypoglycemia and leading to dementia and frailty [20]. Diabetes is associated with frailty and cognitive impairment, both of which make it difficult to maintain glycemic control [21]. Identifying cognitive impairment and frailty is essential in developing appropriate interventions.

The recommended method to reduce diabetes-related complications and costs is to provide education to people on how to manage their condition and maintain glycemic control [1]. Yet, the standard of care is only one diabetes self-management session per year including education on proper nutrition, physical activity, and glucose monitoring [1]. Additional sessions are recommended only when medical complications or major lifestyle transitions occur [22].

When forming a plan of care for older adults with diabetes, evaluation of health status and quality of life need to be undertaken because of the wide variations in physical functioning and medical conditions [23]. This may be why there are so few clinical trials of diet and exercise interventions that specifically target adults aged 65 years and older with type 2 diabetes. Evidence-based recommendations for glycemic control specific for older adults with type 2 diabetes have not been developed, since clinical trials for this population are scarce [23]. For a cohort of *n* = 10,251 adults aged 40–79 with type 2 diabetes, hemoglobin A1c >8.0, and at high risk of cardiovascular disease, intensive therapy (multiple meetings and phone calls) to lower hemoglobin A1c to 6% resulted in higher mortality after 3.7 years compared with the 7-8% standard target, resulting in discontinuation of the treatment in all groups [24].

Concurrent strength and endurance training improved glycemic and hemoglobin A1c control in middle-aged adults with type 2 diabetes [25, 26]. Better glycemic control was achieved by older adults receiving a group behavioral intervention focused in diabetes self-management, including exercise and nutrition compared with individual diabetes education alone [27]. Further evaluation of diabetes education interventions for older adult population is needed, but the existing findings indicate limited effect [27].

## 4. Exercise and Frailty

There is evidence of reduced falls for persons with diabetes who underwent strength, balance, and gait training programs; however, sustainability has not been established [19]. A systematic review of randomized controlled trials of exercise interventions in community-dwelling adults aged 60 years and older demonstrated a 15% greater reduction of falls for the exercise group as compared with the control group [28]. The authors concluded that exercise programs were effective in reducing both the rate of falls and the number of people experience falls [28]. Exercise programs with balance and functional exercises reduced falls, but their effect on other measures of frailty is uncertain. The authors were uncertain of the effects of exercise programs without balance and functional exercises such as walking, dance, and resistance weights, alone on the rate of falls [28]. Less than half of persons with diabetes exercise, and approximately one-quarter meet exercise recommendations [29–31]. Middle-aged outpatients with diabetes had lower physical activity (lower active energy expenditure/day, fewer number steps, and lower physical activity duration) than their matched controls without diabetes [32]. It is likely that this trend would remain the same since physical activity decreases with age. Diabetes combined with physical inactivity accelerates muscle loss in older adults, and the most effective exercise intervention for older adults with diabetes is a combination of resistance and endurance training [33, 34]. For frail, older adults with diabetes and severe functional decline, a multicomponent exercise program is recommended which addresses gait and balance, as well as endurance and power training to counteract functional decline and reduce incidence of falls [33, 35]. For persons with frailty and diabetes, improving functional capacity is equally if not more critical than metabolic control [36].

For older adults with frailty and diabetes, specific guidelines on frequency of exercise performance, repetitions, and progression of intensity for both resistance and endurance training have been proposed [34, 37]. However, attrition can be high and the proportion of older adults that stay engaged in recommended exercise programs has not been determined. In our experience with interventions, attrition rates for older adults ≥40% are common. Frailty was associated with lower hourly measured activity levels in older adults across sex and age-groups [38]. A systematic review of randomized controlled trials (RCTs) of exercise interventions in frail older adults showed that exercise improved physical function [39]. Improvements in lower body strength were found for older adults with type 2 diabetes in a meta-analysis of three RCTs [40]. Lower incidence of frailty was reported for the nutrition and exercise intervention groups versus the control group in a 12-month follow-up of prefrail, community-dwelling older adults [41]. There is a scarcity of RCTs of exercise interventions for frail older adults with type 2 diabetes. Thus, further research is needed in this area.

## 5. Nutrition and Frailty for Older Adults with Type 2 Diabetes

In addition to exercise, nutrition may help reduce the risk for falls; however, nutritional interventions have not been tested as a fall-prevention intervention. Behaviorally focused nutrition education and exercise intervention improved physical function in the MID-FRAIL study, a multinational study of older adults (aged 70 and older) [42]. According to a review, there is insufficient evidence to create nutritional guidelines specific for frail older adults with type 2 diabetes [43]. The limited available research suggests that diets rich in protein and calories may be used to prevent weight loss and malnutrition, but the evidence does not specifically address frailty [43]. Dietary recommendations for persons with frailty include sufficient energy (calories per day determined by age, sex, body weight, height, and physical activity level) and diet quality incorporating foods that are nutritionally dense as opposed to calorie dense [44]. Medical professionals together with dietitians could develop dietary plans specific for the individuals within the wide dietary targets.

Nutritional status, particularly in older adults with type 2 diabetes, may be a confounder in exercise motivation, performance, and results. Unintentional weight loss is one of the five clinical criteria for frailty. Recall that the frailty syndrome requires at least three of the five characteristics: unintentional weight loss, as evidenced by as loss as 5% of body weight from the previous year or 10 lbs; low physical activity, self-reported fatigue, physical slowness, and based on the time to walk 15 feet; in the lowest 20% of grip strength for age, sex, and BMI [5].

Diet has been shown to influence blood glucose regulation and is a key factor in diabetes self-management. Both higher and lower hemoglobin A1c levels were found to be associated with frailty in older adults with type 2 diabetes [20]. There are safety issues for exercising with diabetes; proper nutrition and hydration before, during, and after exercise are essential factors for assuring a safe and pleasant experience. Hypoglycemia can be prevented in older adults with type 2 diabetes by education on exercise timing with medication schedules, meals, and snacks [44, 45]. According to medical nutrition therapy, exercise should be performed postmeal when blood glucose levels are high [46].

According to a position statement by the American Diabetes Association (ADA), low and moderate intensity physical activity should be undertaken by adults with type 2 diabetes, and the risk of exercise-induced adverse events is low [47]. Physical activity and nutritional status have a reciprocal relationship in older adults with diabetes [48]. Physical activity can improve insulin sensitivity, aid in weight maintenance, and increase lean body mass [48]. In turn, physical activity can further the effects of nutrition care with improvements in appetite and glucose control, whereas proper nutrition can be a strategy to increase “energy” and physical activity levels in frail older adults with type 2 diabetes [48]. A review of nine studies of fall interventions for persons with type 2 diabetes and diabetic peripheral neuropathy showed that a targeted, multicomponent program resulted in improved gait and balance without any serious adverse events [49].

## 6. Multimodel Behavioral Interventions for Frailty and Diabetes

How can we motivate and empower older adults with type 2 diabetes to perform the recommended strength and endurance exercises [50] to reduce the rates of frailty and other diabetes complications? There are several behavioral models: cognitive-behavioral therapy, the Health Belief Model, and motivational interviewing have been used to motivate behavioral changes in other populations. *Cognitive-behavioral therapy* is a client-focused technique to increase motivation by removing negative thoughts that interfere in functioning [51]. Feelings of incompetency can interfere with performing exercises, particularly for older adults with comorbidities and/or low physical/functional levels. Cognitive and cognitive-behavioral interventions were more effective in increasing physical activity in older adults than behavioral interventions in a meta-analysis of 20 studies [52]. In cognitive-behavioral therapy, the clinician uses an encouragement approach when the client is ambivalent, whereas in motivational interviewing, a more collaborative strategy, the client would be prompted to discuss their ambivalence.

Another behavioral model used to motivate people to participate in exercise as a treatment for diabetes and frailty is the *Health Belief Model (HBM)* [53]. The HBM was first developed in the 1950s by social psychologists Hochbaum, Rosenstock, and Kegels to explain the reluctance for a free tuberculosis vaccine and this model has been widely applied to other areas of health behaviors. The six constructs of the HBM are (1) perceived susceptibility: person's perception of their likelihood of getting the disease/health condition, (2) perceived severity: the individual's perception of the seriousness of the disease/health condition, (3) perceived benefits, (4) perceived barriers, (5) cues to action (internal, such as a symptom, or external, such as environmental influences), and (6) self-efficacy [54, 55]. The individual's perception of their susceptibility and seriousness of the disease form their perception of threat [54, 55]. According to the HBM, people need to realize that they are vulnerable, understand the severity of their condition and the changes that are required, they need to believe that they can make these changes, and the benefits must outweigh the barriers [54, 55]. Cues to action can serve as motivators to action [56]. Self-efficacy, added as a construct in the 1980s, is an individual's level of confidence in their ability to successfully perform a behavior and is necessary to overcome barriers to take the health action [55, 56].

There are no studies, to date, applying the HBM to prevent frailty and improve diabetes self-management skills in older adults with type 2 diabetes. The majority of studies were in middle-aged to older adults and focused on diabetes self-management skills, only. Significant improvements in self-care behaviors (including diet and exercise) were found applying the HBM to Iranian [57–59], African American women [60], and Pacific Islander populations [57]. Thus, HBM could serve as another possible motivational tool to prevent frailty in older adults with type 2 diabetes.

Another widely used health behavioral model is motivational interviewing (MI); it is suited to those persons who lack motivation to change and can be applied to help individuals increase their physical activity [61, 62]. Motivational interviewing was developed in the clinic to treat addictions and has since been applied to persons with chronic diseases to help them work through their ambivalence about behavior change [63]. There are four core principles of MI: (1) *cultivating change talk*, which engages the client by questions such as “why do you want to change this behavior now?” and “what are you willing to do to change?”; (2) *softening sustain talk* by not paying attention to negative talk; (3) *partnership* where the clinician and client engage in mutual problem solving; and (4) empathy, which the clinician affirms the client's strengths and efforts [64]. The process of MI applied to a specific task can be considered as follows: (a) *engagement*, both the counselor and client establish a helpful connection and working relationship; (b) *focusing*, the counselor maintains the conversation in a specific direction; (c) *evoking*, helps to elicit the client's own motivation for change; and (d) *planning*: this requires a commitment for change along with a specific plan of action [62].

There are no publications applying motivational interviewing to frailty prevention in older adults with type 2 diabetes. Improvements in physical activity have been the consequence of MI; however, these gains are mostly walking at unspecified effort [65]. Studies demonstrating gains in cardio and functional capacity for older adults are limited. What is missing are long-term studies and studies of motivation with strengthening exercises [65]. Motivational interviewing increased physical activity in persons with chronic conditions, according to a meta-analysis of 10 trials. However, there was no evidence that cardiovascular or functional exercise capacity increased [66]. As in the case of cognitive and cognitive-behavioral interventions, motivational interviewing demonstrated increased physical activity, but investigators did not measure functional improvements. Motivational interviewing, alone, may not work with certain racial/ethnic groups who want their physician to prescribe a treatment plan [63]. Based on a systematic review, improvements in diet for persons with diabetes (ages 18 and older) using MI were found in four out of seven trials, whereas the other three showed no improvements [76]. Compared with usual care, group MI was effective in weight loss and improved glycemic index in an 18-month program for women aged approximately 40–60 years with type 2 diabetes [76]. However, African American women compared with White women experienced less weight loss and improvements in glycemic control [67]. There is some evidence to support that motivational approaches work for older adults with various chronic disease conditions [68]. A review of motivational interviewing interventions for lifestyle changes in older adults demonstrated significant improvements in chronic disease management [68]. Motivational interviewing builds on the empowerment model already used in diabetes education. The usefulness of MI on persons with poorly controlled diabetes is currently being investigated in a four-year clinical trial [69].

### 6.1. Novel Motivational Strategy: Combination of HBM and MI to Manage Diabetes and Prevent Frailty

Implementing health behavior change using HBM and MI for clients has not been tested and requires considerable investigation, particularly across race/ethnicity for older populations with type 2 diabetes. We propose a new model combining the HBM and MI aimed at health behavioral change in this population; aspects of this model are already used in diabetes education.  Table 1 shows a nesting of MI within HBM. Increasing self-efficacy may prove to be an effective strategy for long-term behavioral changes in physical activity. Exercise programs for older adults should nurture self-efficacy, the individual's belief that they can achieve the desired results [70].

### 6.2. Considerations of Psychological, Economic and Physiological Barriers for Exercise

Barriers need to be addressed in the application of motivational strategies for persons with type 2 diabetes. Exercise plans need to consider the older adult's access and/or ability to safely exercise in their home, at a community center, or at a park. Older adults of lower socioeconomic status were more likely to perceive their neighborhood as disadvantaged and have lower participation in exercise [72]. Programs recommended by the National Council on Aging provide free classes to members on Medicare and include chair classes. Chair exercises can combine cardio and strength and may be beneficial for adults with orthopedic issues. Motivational techniques for persons with psychological issues such as depression, stress, or anxiety should be done in conjunction with a therapist.

Another barrier to exercise is change in glycemic control. Sedentary older adults with type 2 diabetes who begin an exercise program (either aerobic or strength) will experience noninsulin-dependent uptake of blood glucose because of muscular contractions [47]. Meal timing and medication dose/frequency/timing may need to be adjusted by the physician and dietitian in conjunction with the patient's exercise routine to prevent hypoglycemia. The American Diabetes Association recommends a small carbohydrate snack be carried. Cognitive function may be another barrier to safe exercise. Caretakers and/or family members should be involved in the exercise plan to ensure safety and effectiveness.

## 7. Conclusion

The risk of frailty is doubled in persons with type 2 diabetes, but frailty is not an inevitable consequence of aging and/or diabetes. Older adults with diabetes require preventative interventions to minimize the compounding effects of aging and diabetes on physical function. Health behavioral interventions that motivate and instill confidence have been recommended to make sustainable behavior changes. There is some evidence to support that motivational approaches work for older adults with various chronic disease conditions. However, studies applying motivational strategies to increase physical activity, exercise, improve nutrition and glycemic control, and prevent frailty are lacking for frail older adults with type 2 diabetes. A novel motivational approach was described; it combines aspects of the Health Belief Model and motivational interviewing. Intervention studies incorporating this approach are needed to determine if this client-driven strategy can help various racial/ethnic populations make sustainable health behavior changes by increasing exercise and healthy eating while taking into consideration physiological, psychological, and economic barriers, and finding ways to overcome them.

## Figures and Tables

**Table 1:** Application of motivational strategies and likelihood of following recommendations to manage diabetes and prevent frailty.

HBM construct	MI construct	Modifying factors	Application	Likelihood of action^*∗*^
Perceived susceptibility	Partnership	*Age*, *sex*, *ethnicity*, *personality*, *socioeconomics*, and *knowledge*	Clinician works with client to explain vulnerability consequences: risk of falls for persons with diabetes	Perceived benefits outweigh the perceived barriers
Perceived severity	Partnership/empathy	Perceived threat of falls and uncontrolled diabetes	Clinician educates client about frailty and diabetes in relationship to their individual/social background	Likelihood of performing the recommended exercise and nutrition for diabetes self-management and frailty prevention
Perceived benefits	Cultivating change talk/partnership	*Cues to action* (i) Education (ii) Symptoms (iii) Media	Likelihood of performing the recommended exercise and nutrition for diabetes self-management and frailty prevention (i) Examples of actions: Participating in strength training for older adults at the senior center (ii) Increasing lean protein and vegetables while decreasing bread, rice, and pasta	Clinician indicates specifically how exercise and nutrition can improve the client's quality of life by preventing/reducing frailty and managing blood glucose
Perceived barriers	Softening sustained talk	*Physiological and psychological state (depression*, *stress*, and *anxiety)Hypoglycemia*,*cognitive function*, *fear of falling*, *and safety*		Clinician ignores negative talk and presents solutions
Cues to action	Cultivating change talk/partnership			Clinician provided exercise/nutrition education in conjunction with cognitive, physical and economic limitations (i) Involves physician in medication timing/dose changes (ii) Involves social worker and psychiatrist for economic and psychological issues (iii) Engages client and family/caretakers to discuss what he/she will change and why (iv) Consider structured programs such as SilverSneakers
Self-efficacy	Empathy			Clinician affirms the client's abilities

Adapted from Hamrin et al. [64] and Janz et al. [71] ^*∗*^Likelihood of action is the outcome of constructs, modifying factors and applications.

## References

[1] American Diabetes Association (2019). Improving care and promoting health in populations: standards of medical care in diabetes—2019. *Diabetes Care*.

[2] American Diabetes Association (2019). *Statistics about Diabetes*.

[3] American Diabetes Association (2018). Economic costs of diabetes in the U.S. in 2017. *Diabetes Care*.

[4] Morley J. E., Malmstrom T. K., Rodriguez-Mañas L., Sinclair A. J. (2014). Frailty, sarcopenia and diabetes. *Journal of the American Medical Directors Association*.

[5] Fried L. P., Tangen C. M., Walston J. (2001). Frailty in older adults: evidence for a phenotype. *The Journals of Gerontology Series A: Biological Sciences and Medical Sciences*.

[6] Xue Q.-L. (2011). The frailty syndrome: definition and natural history. *Clinics in Geriatric Medicine*.

[7] Vieira E. R., Mendy A., Prado C. M., Gasana J., Albatineh A. N. (2015). Falls, physical limitations, confusion and memory problems in people with type II diabetes, undiagnosed diabetes and prediabetes, and the influence of vitamins A, D and E. *Journal of Diabetes and its Complications*.

[8] Yang Y., Hu X., Zhang Q., Zou R. (2016). Diabetes mellitus and risk of falls in older adults: a systematic review and meta-analysis. *Age and Ageing*.

[9] Schwartz A. V., Vittinghoff E., Sellmeyer D. E. (2008). Diabetes-related complications, glycemic control, and falls in older adults. *Diabetes Care*.

[10] Vieira E. R., da Silva R. A., Clemson L., Smith M. L., Gu D., Dupre M. (2019). Falls. *Encyclopedia of Gerontology and Population Aging*.

[11] Florence C. S., Bergen G., Atherly A., Burns E., Stevens J., Drake C. (2018). Medical costs of fatal and nonfatal falls in older adults. *Journal of the American Geriatrics Society*.

[12] Centers for Disease Control and Prevention (CDC) (2014). *Costs of Falls Among Older Adults*.

[13] Englander F., Hodson T. J., Terregrossa R. A. (1996). Economic dimensions of slip and fall injuries. *Journal of Forensic Science*.

[14] de Mettelinge T. R., Cambier D., Calders P., Van Den Noortgate N., Delbaere K. (2013). Understanding the relationship between type 2 diabetes mellitus and falls in older adults: a prospective cohort study. *PLoS One*.

[15] Cheng M.-H., Chang S.-F. (2017). Frailty as a risk factor for falls among community dwelling people: evidence from a meta-analysis. *Journal of Nursing Scholarship*.

[16] Nevitt M. C., Cummings S. R., Kidd S., Black D. (1989). Risk factors for recurrent nonsyncopal falls: a prospective study. *JAMA*.

[17] Scott V., Wagar B., Sum A., Metcalfe S., Wagar L. (2010). A public health approach to fall prevention among older persons in Canada. *Clinics in Geriatric Medicine*.

[18] Florida Department of Health (2011). *Florida Injury Facts: Unintentional Falls. Injury Prevention Program*.

[19] Crews R. T., Yalla S. V., Fleischer A. E., Wu S. C. (2013). Growing troubling triad: diabetes, aging, and falls. *Journal of Aging Research*.

[20] Yanase T., Yanagita I., Muta K., Nawata H. (2018). Frailty in elderly diabetes patients. *Endocrine Journal*.

[21] Thein F. S., Li Y., Nyunt M. S. Z., Gao Q., Wee S. L., Ng T. P. (2018). Physical frailty and cognitive impairment is associated with diabetes and adversely impact functional status and mortality. *Postgraduate Medicine*.

[22] Powers M. A., Bardsley J., Cypress M. (2017). Diabetes self-management education and support in type 2 diabetes: a joint position statement of the American diabetes association, the American association of diabetes educators, and the academy of nutrition and dietetics. *The Diabetes Educator*.

[23] Abdelhafiz A. H., Sinclair A. J. (2013). Management of type 2 diabetes in older people. *Diabetes Therapy*.

[24] ACCORD Study Group, Gerstein H. C., Miller M. E. (2011). Long-term effects of intensive glucose lowering on cardiovascular outcomes. *New England Journal of Medicine*.

[25] Church T. S., Blair S. N., Cocreham S. (2010). Effects of aerobic and resistance training on hemoglobin A1c levels in patients with type 2 diabetes: a randomized controlled. *JAMA*.

[26] Cadore E. L., Izquierdo M. (2013). How to simultaneously optimize muscle strength, power, functional capacity, and cardiovascular gains in the elderly: an update. *Age*.

[27] Beverly E. A., Fitzgerald S., Sitnikov L., Ganda O. P., Caballero A. E., Weinger K. (2013). Do older adults aged 60–75 years benefit from diabetes behavioral interventions?. *Diabetes Care*.

[28] Sherrington C., Fairhall N. J., Wallbank G. K. (2019). Exercise for preventing falls in older people living in the community. *Cochrane Database of Systematic Reviews*.

[29] Hamasaki H. (2016). Daily physical activity and type 2 diabetes: a review. *World Journal of Diabetes*.

[30] Peyrot M., Rubin R. R., Lauritzen T., Snoek F. J., Matthews D. R., Skovlund S. E. (2005). Psychosocial problems and barriers to improved diabetes management: results of the cross-national diabetes attitudes, wishes and needs (DAWN) study. *Diabetic Medicine*.

[31] Resnick H. E., Foster G. L., Bardsley J., Ratner R. E. (2006). Achievement of American diabetes association clinical practice recommendations among U.S. adults with diabetes, 1999–2002: the national health and nutrition examination survey. *Diabetes Care*.

[32] Fagour C., Gonzalez C., Pezzino S. (2013). Low physical activity in patients with type 2 diabetes: the role of obesity. *Diabetes & Metabolism*.

[33] Cadore E. L., Izquierdo M. (2015). Exercise interventions in polypathological aging patients that coexist with diabetes mellitus: improving functional status and quality of life. *Age*.

[34] Sinclair A. J., Sinclair H., Bellary S., Rodriguez-Manas L. (2016). The emergence of frailty and sarcopaenia in diabetes mellitus: description of inter-relationships and clinical importance. *Cardiovascular Endocrinology*.

[35] Cadore E. L., Moneo A. B. B., Mensat M. M. (2014). Positive effects of resistance training in frail elderly patients with dementia after long-term physical restraint. *Age*.

[36] Rodríguez-Mañas L., Bayer A. J., Kelly M. (2014). An evaluation of the effectiveness of a multi-modal intervention in frail and pre-frail older people with type 2 diabetes—the MID-Frail study: study protocol for a randomised controlled trial. *Trials*.

[37] Sinclair A. J., Abdelhafiz A., Dunning T. (2017). An international position statement on the management of frailty in diabetes mellitus: summary of recommendations 2017. *The Journal of Frailty and Aging (JFA)*.

[38] Huisingh-Scheetz M., Wroblewski K., Kocherginsky M. (2017). The relationship between physical activity and frailty among U.S. older adults based on hourly accelerometry data. *The Journals of Gerontology: Series A*.

[39] de Labra C., Guimaraes-Pinheiro C., Maseda A., Lorenzo T., Millán-Calenti J. C. (2015). Effects of physical exercise interventions in frail older adults: a systematic review of randomized controlled trials. *BMC Geriatrics*.

[40] Hovanec N., Sawant A., Overend T. J., Petrella R. J., Vandervoort A. A. (2012). Resistance training and older adults with type 2 diabetes mellitus: strength of the evidence. *Journal of Aging Research*.

[41] Serra-Prat M., Sist A., Domenich R. (2017). Effectiveness of an intervention to prevent frailty in pre-frail community-dwelling older people consulting in primary care: a randomised controlled trial. *Age and Ageing*.

[42] Rodriguez-Mañas L., Laosa O., Vellas B. (2019). Effectiveness of a multimodal intervention in functionally impaired older people with type 2 diabetes mellitus. *Journal of Cachexia, Sarcopenia and Muscle*.

[43] Canada Agency for Drugs and Technology in Health (2015). *Diabetic Diets for Frail Elderly Long-Term Care Residents With Type II Diabetes Mellitus: A Review of Guidelines [Internet]*.

[44] Lorenzo-López L., Maseda A., de Labra C., Regueiro-Folgueira L., Rodríguez-Villamil J. L., Millán-Calenti J. C. (2017). Nutritional determinants of frailty in older adults: a systematic review. *BMC Geriatrics*.

[45] Ekong G., Kavookjian J. (2016). Motivational interviewing and outcomes in adults with type 2 diabetes: a systematic review. *Patient Education and Counseling*.

[46] Gray A., Feingold K. R., Anawalt B., Boyce A. (2000). Nutritional recommendations for individuals with diabetes. *Endotext [Internet]*.

[47] Colberg S. R., Sigal R. J., Yardley J. E. (2016). Physical activity/exercise and diabetes: a position statement of the American diabetes association. *Diabetes Care*.

[48] Stanley K. (2014). Nutrition considerations for the growing population of older adults with diabetes. *Diabetes Spectrum*.

[49] Gu Y., Dennis S. M. (2017). Are falls prevention programs effective at reducing the risk factors for falls in people with type-2 diabetes mellitus and peripheral neuropathy: a systematic review with narrative synthesis. *Journal of Diabetes and its Complications*.

[50] Department of Health and Human Services U.S. (2018). *Physical Activity Guidelines for Americans*.

[51] Beck J. S., Beck A. (2011). *Cognitive Behavioral Theory*.

[52] Chase J.-A. D. (2013). Physical activity interventions among older adults: a literature review. *Research and Theory for Nursing Practice*.

[53] Green E. C., Murphy E. (2014). Health belief model. *The Wiley Blackwell Encyclopedia of Health, Illness, Behavior, and Society*.

[54] Rosenstock I. M. (1974). Historical origins of the health belief model. *Health Education and BehaviorHealth Education Monographs*.

[55] Champion V. L., Skinner C. S., Glanz K., Rimer B. K., Viswanath K. (2008). The health belief model. *Health Behavior and Health Education*.

[56] McElfish P. A., Hallgren E., Henry L. J., Ritok M., Rubon-Chutaro J., Kohler P. (2016). Health Beliefs of Marshallese regarding type 2 diabetes. *American Journal of Health Behavior*.

[57] Bayat F., Shojaeezadeh D., Baikpour M., Heshmat R., Baikpour M., Hosseini M. (2013). The effects of education based on extended health belief model in type 2 diabetic patients: a randomized controlled trial. *Journal of Diabetes & Metabolic Disorders*.

[58] Dehghani-Tafti A., Mazloomy Mahmoodabad S. S., Morowatisharifabad M. A., Afkhami Ardakani M., Rezaeipandari H., Lotfi M. H. (2015). Determinants of self-care in diabetic patients based on health belief model. *Global Journal of Health Science*.

[59] Shabibi P., Abedzadeh Zavareh M. S., Sayehmiri K. (2017). Effect of educational intervention based on the health belief model on promoting self-care behaviors of type-2 diabetes patients. *Electronic Physician*.

[60] Koch J. (2002). The role of exercise in the African-American woman with type 2 diabetes mellitus: application of the health belief model. *Journal of the American Academy of Nurse Practitioners*.

[61] Hardcastle S. J., Hancox J., Hattar A., Maxwell-Smith C., Thøgersen-Ntoumani C., Hagger M. S. (2015). Motivating the unmotivated: how can health behavior be changed in those unwilling to change?. *Frontiers in Psychology*.

[62] Miller W. R., Rollnick S. (2013). *Motivational Interviewing: Helping People Change*.

[63] Resnicow K., McMaster F. (2012). Motivational interviewing: moving from why to how with autonomy support. *International Journal of Behavioral Nutrition and Physical Activity*.

[64] Hamrin V., Sinclair V. G., Gardner V. (2017). Theoretical approaches to enhancing motivation for adherence to antidepressant medications. *Archives of Psychiatric Nursing*.

[65] Bennett J. A., Winters-Stone K. (2011). Motivating older adults to exercise: what works?. *Age and Ageing*.

[66] O’Halloran P. D., Blackstock F., Shields N. (2014). Motivational interviewing to increase physical activity in people with chronic health conditions: a systematic review and meta-analysis. *Clinical Rehabilitation*.

[67] Smith Y. R., Johnson A. M., Newman L. A., Greene A., Johnson T. R. B., Rogers J. L. (2007). Perceptions of clinical research participation among African American women. *Journal of Women’s Health*.

[68] Cummings S. M., Cooper R. L., Cassie K. M. (2009). Motivational interviewing to affect behavioral change in older adults. *Research on Social Work Practice*.

[69] Welch G., Rose G., Ernst D. (2006). Motivational interviewing and diabetes: what is it, how is it used, and does it work?. *Diabetes Spectrum*.

[70] Rivera-Torres S., Fahey T. D., Rivera M. A. (2019). Adherence to exercise programs in older adults: informative report. *Gerontology & Geriatric Medicine*.

[71] Janz N. K., Champion V. L., Strecher V. J., Glanz K., Rimer B. K., Lewis F. M. (2002). The health belief model. *Health Behavior and Health Education: Theory, Research and Practice*.

[72] Wee L. E., Tsang Y. Y. T., Tay S. M. (2019). Perceived neighborhood environment and its association with health screening and exercise participation amongst low-income public rental flat residents in Singapore. *International Journal of Environmental Research and Public Health*.

